# Case Report: A rare case of IgG4-related disease initially manifesting with diarrhea and jaundice

**DOI:** 10.3389/fimmu.2026.1893575

**Published:** 2026-07-28

**Authors:** Juan Gao, Wei Qi, Xin Wang, Nan Wang, Ruo-Lin Han, Zhi-Juan Gu, Zhuo Li

**Affiliations:** 1 Department of Clinical Laboratory, The First Affiliated Hospital of Xi’an Medical University, Xi’an, Shaanxi, China; 2 Ward 1, Department of Gastroenterology, The First Affiliated Hospital of Xi’an Medical University, Xi’an, Shaanxi, China; 3 Ward 1, Department of General Surgery, The First Affiliated Hospital of Xi’an Medical University, Xi’an, Shaanxi, China; 4 Department of Imaging, The First Affiliated Hospital of Xi’an Medical University, Xi’an, Shaanxi, China

**Keywords:** cholangitis, diarrhea, immunoglobulin G4 (IgG4), jaundice, pancreatitis

## Abstract

**Background:**

IgG4-related disease (IgG4-RD) is a systemic immune-mediated fibrosing inflammatory disorder with multi-organ involvement. Due to highly variable and nonspecific clinical manifestations, atypical cases are prone to misdiagnosis and missed diagnosis. This study reports an IgG4-RD case presenting with rare initial symptoms of diarrhea and jaundice, aiming to enrich the clinical spectrum of atypical IgG4-RD and improve early differential diagnosis.

**Case presentation:**

A 62-year-old male with well-controlled type 2 diabetes mellitus presented with 10 days of refractory mucoid diarrhea with occasional hematochezia and 2 days of progressive obstructive jaundice, pale stool, and generalized pruritus, accompanied by a 25 kg unintentional weight loss within one year. Laboratory tests showed markedly elevated transaminases, bilirubin, bile acid, alkaline phosphatase, gamma-glutamyl transpeptidase, CA19-9, erythrocyte sedimentation rate, and serum IgG4. Imaging revealed extrahepatic bile duct wall thickening and stenosis, sausage-like pancreatic swelling, and multi-organ lesions involving the bilateral renal hilum and retroperitoneum. Since pathological biopsy was declined by the patient and his family, a clinical suspicion of IgG4-related sclerosing cholangitis was established based on consistent clinical, serological, and typical multi-organ imaging features, although histopathology remains the gold standard for definitive diagnosis. The patient received liver-protective, glucocorticoid anti-inflammatory, nutritional, and anti-osteoporosis treatments. His symptoms resolved rapidly with significantly improved liver function, and he was discharged in stable condition. Ten-month regular follow-up was completed after discharge; no disease recurrence or adverse reactions were observed during gradual steroid tapering and maintenance phase.

**Conclusion:**

IgG4-RD with initial diarrhea and jaundice is easily misdiagnosed as cholangiocarcinoma or primary sclerosing cholangitis. Markedly elevated serum IgG4 combined with characteristic multi-organ imaging changes can strongly support a clinical suspicion of IgG4-RD, but cannot independently establish a definitive diagnosis without histopathological evidence. The gold-standard definite diagnosis requires integrated fulfillment of clinical, serological, radiological and pathological criteria. Glucocorticoid therapy is effective for symptom relief and biochemical improvement in suspected cases. The patient maintained sustained remission during the 10-month follow-up without relapse, demonstrating favorable medium-term prognosis, while long-term serial monitoring is still essential to further reduce late recurrence risk and optimize long-term prognosis.

## 1 Introduction

IgG4-related disease (IgG4-RD) is a systemic immune-mediated fibrosing inflammatory disorder that can involve multiple organs. Its pathogenesis remains unclear. The main histopathological features include infiltration of lymphocytes and plasma cells predominantly composed of IgG4-positive plasma cells, accompanied by storiform fibrosis, obliterative phlebitis and eosinophilic infiltration (1, 2). This disease triggers chronic immune activation, leading to tissue damage dominated by fibrosis (3). IgG4-SC is a rare autoimmune biliary disorder characterized by elevated serum IgG4 levels, extensive infiltration of IgG4-positive plasma cells, and progressive biliary tract fibrosis (4–6). It often mimics biliary malignancy clinically, easily causing misdiagnosis and requiring combined imaging, serology and pathology for accurate identification.

Current consensus diagnostic criteria for IgG4-RD uniformly regard histopathological examination as the mandatory gold standard for definitive diagnosis. Serum IgG4 elevation and typical multi-organ imaging manifestations are critical auxiliary clues, yet neither biomarker nor radiological findings alone or in combination can replace pathological confirmation to reach a definitive diagnosis.

This case reports a patient with IgG4-RD who presented with diarrhea and scleral and cutaneous jaundice. Although the patient refused pathological biopsy, a clinical suspicion of IgG4-RD was still established according to markedly elevated serum IgG4 levels and characteristic imaging manifestations of multi-organ involvement, including the bile ducts, pancreas, bilateral kidneys and retroperitoneum. After glucocorticoid treatment, the patient’s symptoms and liver function were improved, and sustained remission was maintained throughout the subsequent 10-month follow-up period without any disease flare. This case indicates that when pathological evidence is unavailable in clinical practice, the combination of serological and imaging features can serve as an important auxiliary basis for suspected IgG4-RD diagnosis to help reduce misdiagnosis and missed diagnosis, but diagnostic certainty must be appropriately limited without histology. This report provides a reference for the diagnosis and management of similar biopsy-unavailable cases.

## 2 Case presentation

Medical history: On August 9, 2025, a 62-year-old male patient presented with diarrhea of unknown cause 10 days prior, with a maximum of 10 bowel movements daily. The stool was mucoid with occasional fresh blood. Two days ago, he developed jaundice of the skin and sclera, pale-colored stool and pruritus, without abdominal distension, abdominal pain, chest tightness or shortness of breath. Hepato-biliary-pancreatic-splenic ultrasonography revealed dilatation of intrahepatic and extrahepatic bile ducts, thickening of the upper common bile duct wall, and hypoechoic lesion in the common bile duct closely related to the pancreatic head, suggestive of space-occupying lesion, further examination was recommended. He was admitted to the General Surgery Department of our hospital with the diagnosis of obstructive jaundice. During the course of illness, the patient was conscious with fair mental status, poor appetite and sleep, dark yellow urine, and had a body weight loss of approximately 25 kg in the past year.

Past medical history: The patient had a 2-year history of diabetes mellitus, regularly taking Dapagliflozin with well-controlled blood glucose. He had a previous history of abdominal burn injury with good recovery. He was hospitalized in the Pain Department of our hospital for left leg herpes zoster two months ago, and the condition was not fully cured.

Physical examination: Temperature: 36.5°C, Pulse: 78 beats/min, Respiration: 18 breaths/min, Blood pressure: 124/78 mmHg. Jaundice was noted on the skin, mucous membranes and sclera. Extensive fibrous scars were seen in the lower abdomen. The abdomen was soft without tenderness or rebound tenderness, and no abdominal mass was palpable. The liver was not palpable below the costal margin, and Murphy’s sign was negative. No other obvious abnormalities were found.

Laboratory tests on August 9, 2025: Total bilirubin: 82.00 μmol/L, Direct bilirubin: 51.90 μmol/L, Indirect bilirubin:30.10 μmol/L, Total bile acid:478.80 μmol/L, Aspartate aminotransferase: 84.40 U/L, Alanine aminotransferase:137.80 U/L, Alkaline phosphatase: 227.50 U/L, Gamma-glutamyl transpeptidase:325.00 U/L; Albumin:33.5 g/L, Globulin:50.8 g/L, Albumin/Globulin ratio: 0.7; Blood glucose: 6.58 mmol/L; Red blood cell count: 3.84×10¹²/L, Hemoglobin: 122 g/L; CA19-9: 214 U/mL, EBV-VCA-IgG:50 AU/mL, EBV-NA-IgG: 36 AU/mL; Urine bilirubin: 2+, Urine glucose: 4+(**Table 1**).

**Table 1:** Laboratory test results of the patient (different time points).

Parameter	Recorded value (8.9)	Recorded value (8.15)	Recorded value (8.22)	Standard value
Total bilirubin	82.00	68.30	39.20	0-26 μmol/L
Direct Bilirubin	51.90	41.80	20.80	0-4 μmol/L
Indirect Bilirubin	30.10	26.50	18.40	0-20 μmol/L
Total Bile Acid	478.80	307.60	71.80	0-15 μmol/L
Aspartate Aminotransferase	84.40	60.30	43.80	15–40 U/L
Alanine Aminotransferase	137.80	71.00	56.90	9–50 U/L
Alkaline Phosphatase	227.50	159.90	117.70	45–125 U/L
Gamma-Glutamyl Transferase	325.00	142.20	60.40	10–60 U/L
Albumin	33.5	32.8	29.6	40–55 g/L
Globulin	50.8	16.4	40	20–40 g/L
A/G	0.7	0.7	0.7	1.2-2.4
Glucose	6.58	/	/	3.9-6.1mmo1/L
Red blood cell	3.84	/	/	4.3-5.8 10^12/L
Hemoglobin	122	/	/	130–175 g/L
Carbohydrate Antigen 19-9	214	/	72.9	≤30 U/mL
EBV-VCA-IgG	50	/	/	0–2 AU/mL
EBV-NA-IgG	36	/	/	0–2 AU/mL
Urine Bilirubin	2+	/	/	–
Urine Glucose:	4+	/	/	–
Immunoglobulin G4	/	37.100	/	≤2 g/L
Erythrocyte Sedimentation Rate	/	81	/	0–15 mm/h

Abdominal ultrasonography on August 10, 2025: Dilatation of intrahepatic and extrahepatic bile ducts, thickening of the upper common bile duct wall; hypoechoic lesion in the common bile duct adjacent to the pancreatic head, suspected space-occupying lesion, further examination suggested. Intrahepatic calcification foci were detected. The gallbladder was enlarged with rough and thickened wall and cholesterol crystal deposition on gallbladder wall. The pancreas was enlarged with decreased echo and dilatation of main pancreatic duct. No obvious abnormality was found in the spleen.

Preliminary Diagnosis based on symptoms, physical signs and auxiliary examinations: 1.Obstructive jaundice; 2.Type 2 diabetes mellitus; 3.Space-occupying lesion of bile duct. Further examinations including plain and contrast-enhanced abdominal CT, as well as contrast-enhanced MRCP of liver, gallbladder, pancreas and spleen shall be completed to confirm the nature of the lesion.

**Figure 1:**
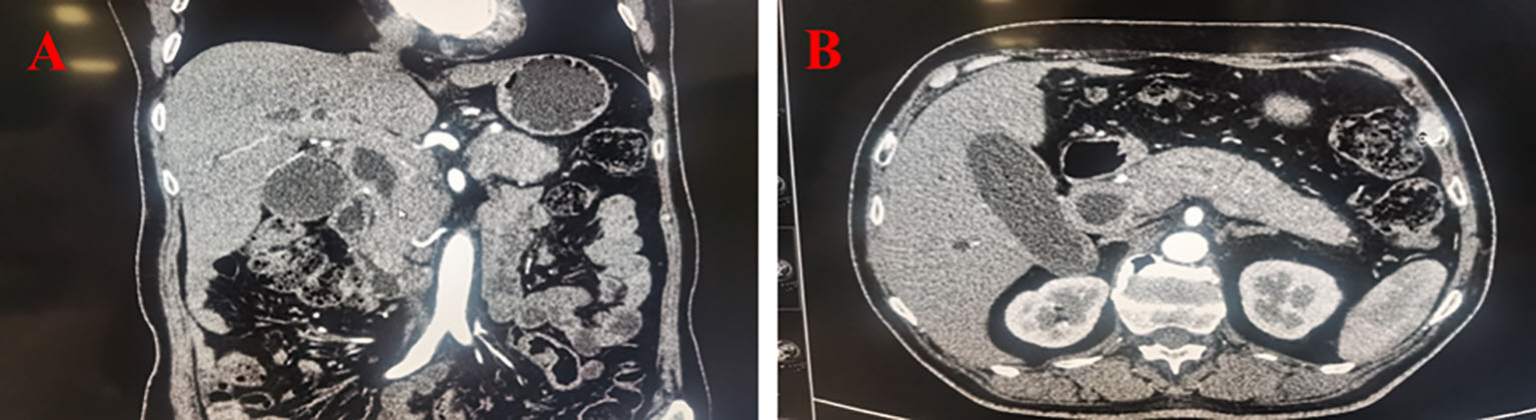
Plain CT plus three-dimensional reconstruction of upper abdomen, lower abdomen and pelvic cavity (transverse, coronal and sagittal planes). **(A)** Significant circumferential thickening of extrahepatic bile duct wall, most prominent at hepatic hilum and pancreatic segment, with corresponding luminal stenosis and dilatation of residual intrahepatic and extrahepatic bile ducts. **(B)** Pancreatic swelling presenting as sausage-like change, predominantly in pancreatic head, with rough pancreatic capsule and slight peripheral exudation.

On August 11, 2025, the patient underwent 3T plain MRI combined with DWI, contrast-enhanced scanning and MRCP of the liver, gallbladder, pancreas and spleen. The results indicated: 1.Marked circumferential thickening of the extrahepatic bile duct wall with luminal stenosis at the hepatic hilum and pancreatic segment, accompanied by dilatation of the remaining intrahepatic and extrahepatic bile ducts, suggestive of sclerosing cholangitis; 2.Pancreatic swelling with sausage-like appearance and rough pancreatic capsule, highly suggestive of autoimmune pancreatitis; 3.Massive irregular soft tissue shadows surrounding the renal pelvis at bilateral renal hilum, as well as nodular soft tissue thickening anterior to the T9–10 vertebral bodies. Laboratory tests are recommended to rule out IgG4-related diseases (**Figure 1**).

Laboratory tests on August 15, 2025: IgG4: 37.10 g/L, ESR: 81 mm/h. No abnormalities were found in autoantibody spectrum, five items of vasculitis-related antibodies and autoimmune liver disease related tests.

Referring to the diagnostic criteria in the Chinese Expert Consensus on Diagnosis and Treatment of IgG4-Related Diseases (7), combined with the patient’s symptoms of obstructive jaundice, markedly elevated serum IgG4 level, and imaging manifestations showing multi-site involvement of retroperitoneal tissues, bile ducts, pancreas and bilateral kidneys, IgG4-related disease is highly suspected clinically. Histopathological examination is the core evidence for definite diagnosis of this disease, yet puncture biopsy was refused by the patient and his family members. In view of typical clinical manifestations, characteristic imaging findings, significant increase of serum IgG4 and exclusion of other similar diseases, this case meets the criteria for suspected diagnosis. Final diagnoses: 1.IgG4-related cholangitis; 2.IgG4-related disease; 3.autoimmune pancreatitis.

After a definite diagnosis of IgG4-related disease was established, the patient was prescribed prednisone acetate tablets 30 mg once daily in the morning, calcium carbonate and vitamin D3 granules one sachet 1–2 times daily, and ursodeoxycholic acid tablets one tablet twice daily. One week after treatment, the patient’s skin pruritus and jaundice improved obviously, with no abdominal pain, diarrhea, hematochezia, fever, chills or other discomforts. Reexamination showed total bilirubin 39.20 μmol/L, direct bilirubin 20.80 μmol/L, total bile acid 71.80 μmol/L, aspartate aminotransferase 43.80 U/L, alanine aminotransferase 56.90 U/L, gamma-glutamyl transpeptidase 60.40 U/L, and CA19-9 72.9 U/ml (**Table 1**). Liver function was ameliorated compared with baseline, and the patient’s condition remained stable. At the 10 months follow-up, the patient was asymptomatic. Prednisone acetate was gradually tapered by 5 mg every 1–2 weeks. No disease recurrence occurred during dose reduction, and no immunosuppressive agents were administered throughout the tapering period.

## 3 Discussion

IgG4-RD frequently invades multiple organs, with the pancreas as the most common lesion site, followed by bile ducts, salivary glands, kidneys and retroperitoneal tissues (8). Its subtype IgG4-SC is characterized by obstructive jaundice, biliary wall thickening and systemic fibroinflammation, yet vague early symptoms often lead to missed or misdiagnosis (9, 10). Typical manifestations include painless progressive jaundice, intractable pruritus, tea-colored urine and clay stool; some patients merely show fatigue, weight loss or incidental liver dysfunction on physical examination (9). IgG4-SC shares overlapping manifestations with cholangiocarcinoma, PSC and biliary stones, demanding rigorous differential diagnosis (11–13). It is frequently combined with autoimmune pancreatitis and extra-pancreatic lesions such as Mikulicz disease and IgG4-related nephropathy (10, 12–14).

Definite IgG4-SC diagnosis relies on three core pillars as specified in the 2020 Japanese criteria, 2019 ACR/EULAR classification and Chinese IgG4-RD guidelines (15): multi-organ fibroinflammatory lesions, elevated serum IgG4 and characteristic histopathology, none of which can independently establish diagnosis. Serum IgG4 ≥gG4 mg/dL serves as a screening marker, while a 200 mg/dL cutoff improves specificity (16). Nevertheless, this biomarker has limitations: mild elevation occurs in PSC, cholangiocarcinoma and chronic pancreatitis, and 10%–30% pathologically confirmed IgG4-RD patients retain normal IgG4 levels, so it cannot act as a standalone diagnostic index. Histopathology remains the gold standard, defined by >10 IgG4-positive plasma cells per high-power field, IgG4-positive/IgG ratio >40%, storiform fibrosis and obliterative phlebitis (17, 18). Raising the cellular threshold to 50 cells helps exclude malignancy, and preoperative biopsy effectively avoids unnecessary surgery misdiagnosed as biliary carcinoma. Glucocorticoids achieve an induction remission rate of 99.5%, rapidly improving bilirubin and ALP within two weeks (19, 20).

This case has an obvious limitation: biopsy was rejected by the patient, lacking pathological gold-standard evidence. Extremely elevated serum IgG4 (37.10 g/L) and typical multi-organ imaging only support high-probability clinical suspicion rather than definitive diagnosis. Clinicians should avoid overinterpreting serology and imaging results without histological confirmation, as isolated IgG4 elevation and similar radiological signs cannot fully rule out biliary malignancies.

Cholangiocarcinoma and PSC are top differential diagnoses for patients with pancreatic enlargement and obstructive jaundice. Cholangiocarcinoma features abrupt biliary cutoff, peripancreatic vascular invasion, markedly elevated CA19–9 and no response to steroids, without diffuse pancreatic swelling or multi-organ retroperitoneal lesions. Most PSC patients are young and complicated with inflammatory bowel disease, presenting segmental intrahepatic strictures and normal serum IgG4 without systemic fibrotic lesions. Other alternatives including chronic cholangitis and pancreatic cancer can be excluded via combined serology, imaging and therapeutic response observation.

Glucocorticoids serve as first-line induction therapy to suppress plasma cell infiltration and fibrosis. Standard prednisone doses (0.6–1.0 mg/kg daily) rapidly relieve cholestasis, as evidenced by the patient’s liver function recovery within one week. Slow dose tapering prevents disease flare-ups, with calcium and vitamin D3 routinely supplemented to avoid steroid-induced osteoporosis. The 10-month follow-up confirmed sustained complete remission without relapse or adverse drug reactions, verifying reliable medium-term efficacy of standardized steroid regimens.

Early identification of atypical IgG4-RD presentations bears great clinical significance. Most patients initially develop painless jaundice or recurrent pancreatitis, while diarrhea as the prodromal symptom is rare and easily misdiagnosed as intestinal disorders before biliary obstruction emerges. For patients with unexplained chronic diarrhea followed by obstructive jaundice, timely serum IgG4 testing and full abdominal multi-phase CT/MRI facilitate differentiation between treatable IgG4-RD and incurable biliary malignancies, preventing redundant surgical intervention.

This report describes an IgG4-RD case with initial diarrhea and jaundice. Without pathological biopsy, high-probability diagnosis was reached based on drastically elevated IgG4 and typical imaging consistent with Chinese expert consensus (7). Steroids rapidly alleviated jaundice and pruritus and improved liver function. Ten-month continuous follow-up confirmed persistent remission, further supporting steroid efficacy. Clinicians must include IgG4-RD in differential diagnoses for obstructive jaundice with multi-system lesions, and perform serum IgG4 and comprehensive imaging screening to reduce misdiagnosis. Long-term serial monitoring of serum IgG4, liver function and abdominal imaging will be scheduled for this patient to track long-term disease activity and late recurrence risk.

## Data Availability

The original contributions presented in the study are included in the article/supplementary material. Further inquiries can be directed to the corresponding author.
